# Enhanced Human Tissue Microdialysis Using Hydroxypropyl-ß-Cyclodextrin as Molecular Carrier

**DOI:** 10.1371/journal.pone.0060628

**Published:** 2013-04-05

**Authors:** Marcus May, Sandor Batkai, Alexander A. Zoerner, Dimitrios Tsikas, Jens Jordan, Stefan Engeli

**Affiliations:** 1 Institute of Clinical Pharmacology, Hannover Medical School, Hannover, Germany; 2 Institute for Molecular and Translational, Hannover Medical School, Hannover, Germany; Centre of Marine Sciences & University of Algarve, Portugal

## Abstract

Microdialysis sampling of lipophilic molecules in human tissues is challenging because protein binding and adhesion to the membrane limit recovery. Hydroxypropyl-ß-cyclodextrin (HP-ß-CD) forms complexes with hydrophobic molecules thereby improving microdialysis recovery of lipophilic molecules *in vitro* and in rodents. We tested the approach in human subjects. First, we determined HP-ß-CD influences on metabolite stability, delivery, and recovery *in vitro*. Then, we evaluated HP-ß-CD as microdialysis perfusion fluid supplement in 20 healthy volunteers. We placed 20 kDa microdialysis catheters in subcutaneous abdominal adipose tissue and in the vastus lateralis muscle. We perfused catheters with lactate free Ringer solution with or without 10% HP-ß-CD at flow rates of 0.3–2.0 µl/min. We assessed tissue metabolites, ultrafiltration effects, and blood flow. In both tissues, metabolite concentrations with Ringer+HP-ß-CD perfusate were equal or higher compared to Ringer alone. Addition of HP-ß-CD increased dialysate volume by 10%. Adverse local or systemic reactions to HP-ß-CD did not occur and analytical methods were not disturbed. HP-ß-CD addition allowed to measure interstitial anandamide concentrations, a highly lipophilic endogenous molecule. Our findings suggest that HP-ß-CD is a suitable supplement in clinical microdialysis to enhance recovery of lipophilic molecules from human interstitial fluid.

## Introduction

Microdialysis is widely applied in basic and clinical research to evaluate interstitial concentrations of endogenous molecules in brain, skin, adipose tissue, skeletal muscle, heart, kidney and liver [1]–[5]. The methodology is also useful to assess tissues concentrations of medications in pharmacokinetic investigations [6], [7]. The main limitation of the technique is that recovery from the interstitial space is profoundly affected by molecular size and physicochemical properties of the analyte. Smaller water soluble molecules such as glucose, pyruvate, lactate, glycerol, and urea show relatively high recoveries and can easily be measured in human tissues. In contrast, microdialysis recovery of larger and lipophilic molecules such as insulin, inflammatory mediators, and lipids is difficult [2], [5]. We reasoned that addition of cyclodextrins to the perfusate could improve recovery of lipophyilic analytes in man. Cyclodextrins are cyclic oligosaccharides forming water soluble inclusion complexes with lipophilic molecules. Hydroxypropyl-β-cyclodextrin (HP-ß-CD) is characterized by good inclusion complexation properties [8] and is considered as safe for human use [9]. In animals, HP-ß-CD enhanced microdialysis recovery of lipophilic molecules [10], [11]. Endocannabinoid sampling efficiency increased 10-fold when HP-ß-CD was added to microdialysis perfusates [12]. We recently studied *in vitro* recovery and delivery of HP-ß-CD and anandamide, and determined the most appropriate HP-ß-CD-enhanced microdialysis protocol for endocannabinoid measurements [13], [14]. Now, we evaluated characteristics of 10% (w/v) HP-ß-CD as an additive to microdialysis perfusates in human adipose tissue and skeletal muscle microdialysis, and demonstrated the feasibility of endocannabinoid microdialysis in human adipose tissue with HP-ß-CD. We monitored tissue metabolites and ethanol recovery to exclude that HP-ß-CD affects glucose metabolism, lipolysis, or blood flow.

## Methods

### Materials

We applied CMA 60 microdialysis catheters with poly aryl ether sulphone (PAES) membranes and 20 kDa cutoff for all experiments. All catheters and pumps, the metabolite analyzer, standard analytes for *in vitro* purposes (glucose, lactate, glycerol, pyruvate and urea), and the sterile perfusion fluid for *in vivo* microdialysis (“T1”: lactate-free Ringer solution containing NaCl 147 mmol/l, KCl 4 mmol/l, CaCl2 2.3 mmol/l, 290 mOsm/kg) were from CMA/µDialysis, Stockholm, Sweden. Sterile Ringer’s solution for *in vitro* experiments was obtained from B. Braun (Melsungen, Germany) and fatty acid free human serum albumin was purchased from Sigma-Aldrich (Steinheim, Germany). HP-ß-CD was purchased as Cavitron W7 HP5 (in compliance with Ph.Eur. 6, Hydroxypropylbetadex) from ISP Fine Chemicals (Assonet, MA, USA). Ringer solution supplemented with HP-ß-CD (10% w/v) was prepared under aseptic conditions and aliquots (10 mL) of the final solution were sterilized in glass bottles at 121°C in the Hannover Medical School Pharmacy. Sterile ethanol in water (95%) (B. Braun, Melsungen, Germany) was added to the perfusion fluid to a concentration of 50 mM immediately before the experiments were begun. We used CMA 107 microdialysis pumps in clinical studies and CMA 402 microdialysis pumps for *in vitro* experiments.

### Analytical Procedures

Microdialysis samples were analyzed for glucose, lactate, pyruvate, glycerol and urea concentrations using the automated enzyme-linked spectrophotometric CMA 600 analyzer [15]. Glucose and Glycerol reveal information regarding glucose supply and lipolysis, respectively [16]–[18]. Lactate and Pyruvate are useful to evaluate glycolysis and oxidative glucose metabolism at the tissue level [3], [4], [19]. Changes in blood flow were determined using the ethanol escape technique, and by estimation of microdialysis urea clearance [20], [21]. Ethanol concentration was measured in perfusate and dialysate using the standard enzymatic assay with alcohol dehydrogenase (Sigma-Aldrich, Seelze, Germany) and a plate reader (Tecan, Infinite F200, Switzerland) as described before [22]. A decrease in the dialysate/perfusate ethanol ratio (“ethanol escape”) described an increase in blood flow and *vice versa* [20]. An increase in urea concentration indicated an increase in blood flow [21]. Ultrafiltration was determined by weighing microdialysis samples that included defined fluid volumes according to pump flow rates. Before the experiments the weight of the empty vials was determined by a special accuracy scale (Sartorius Analytical, Goettingen, Germany) and ultrafiltration was revealed by weight differences before and after microdialysis. Anandamide and HP-ß-CD were measured by LC-MS/MS protocols as previously described [13], [14]. Statistical analysis was performed using GraphPad Prism Version 5 (GraphPad Software Inc., La Colla, CA, USA). All values are mean±SEM unless stated otherwise.

### In vitro Experiments

A custom made *in vitro* microdialysis system (Prof. Kloft, University Halle-Wittenberg, patent pending) was used. Experiments were performed under conditions mimicking *in vivo* conditions with the matrix fluid (15 mL) kept at 37°C and containing fatty acid free human serum albumin (3.5 g/l). Solutions of the following substances, referred to as metabolites, were prepared at physiological concentrations unless otherwise noted: glucose and lactate were used at 6 mmol/l each, pyruate at 200 µmol/l, glycerol at 500 µmol/l and urea at 10 mmol/l. In each experiment, perfusion fluids with and without 10% HP-ß-CD (w/v) were compared. Experiments were repeated twice unless otherwise stated.

For stability experiments, the catheter was placed in matrix fluid (37°C) matching the perfusion fluid - with exception of the HP-ß-CD content - such that a negligible metabolite net flow across the catheter’s membrane was expected. Metabolite concentrations in perfusion and matrix fluids were in a physiologically relevant range. Experiments were performed with a perfusate flow rate of 1 µL/min over 3 h following 30 minutes equilibration time. The microdialysate sampling interval was 10 minutes. At the beginning and at the end of the experiments, aliquots of perfusion and matrix fluids (each 10 µl) were analyzed.

For delivery experiments, perfusion fluid was prepared with physiological metabolite concentrations. The catheter was placed in matrix fluid at 37°C without any dissolved metabolites. Relative recovery of the delivery experiments was measured according to the formula:
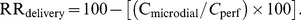
(1)


A flow rate of 2 µL/min was chosen because microdialysate metabolite concentrations were below the limit of quantification with 1 µL/min flow rate. Again, microdialysates were collected every 10 min.

Recovery experiments were performed by delivering perfusion fluid without metabolites at 0.3 µL/min, 1 µL/min, and 2 µL/min through a catheter placed in matrix fluid at 37°C containing metabolites at physiological concentrations as described above. The corresponding sampling intervals were 33, 10 and 5 min such that each microdialysis sample comprised approximately 10 µL. Both, in matrix fluid and in microdialysis samples, metabolites were measured. Relative recovery of recovery experiments was calculated by the formula:
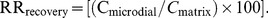
(2)


### Clinical Studies

Twenty healthy volunteers were included in the study. Written informed consent was provided by all participants before being enrolled. The study protocol was reviewed and approved by the Ethics Committee of Hannover Medical School. Following informed consent, demographic data, medical history, and concomitant medication were assessed. All participants were non-smokers and had no history of allergies, gastrointestinal, endocrinological, or psychiatric disorders, and presented with normal physical exams, blood pressure and electrocardiograms. Blood tests confirmed normal blood count and normal liver and renal functions.

All subjects were studied while resting in supine position on a comfortable bed in a room kept at 23 to 25°C over a time period of six hours. After overnight fasting, a venous catheter was placed in the antecubital vein. At least two microdialysis catheters were placed under sterile conditions into adipose tissue (8 to 10 cm left of the umbilicus) and into the lower third of the *vastus lateralis* muscle and connected to a CMA 107 microdialysis pump. 12 participants were instrumented with four catheters, two in adipose tissue and two in skeletal muscle. After probe insertion, catheters were perfused in a parallel fashion with sterile Ringer including 50 mM ethanol solution with or without 10% HP-ß-CD (w/v). After a sufficient equilibration period, the first microdialysis samples were collected. During the experiments, venous blood was drawn from all volunteers, samples were immediately centrifuged at 3500 rev/min for 10 minutes, and the supernatants were separated. Plasma and microdialysis samples were placed on ice immediately after collection and stored at −80°C until analysis.

Dependency of metabolite relative recovery on perfusion flow rate and “ethanol escape” was determined in adipose tissue and skeletal muscle using 0.3 µL/min, 0.5 µl/min, 1 µL/min and 2 µL/min flow rates. The theoretical interstitial metabolite concentration was calculated using the method of flow rate variation [23]. Relative recovery is described by the asymptotic exponential function:
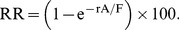
(3)


Relative recovery at a flow rate of “zero” µl/min is considered to be 100%, and thus the measured microdialysate concentration equal to the interstitial metabolite tissue concentration. By nonlinear regression and extrapolation to zero of the above mentioned exponential formula F3, intracellular metabolite concentration was estimated. Relative recovery was calculated with the estimated tissue concentrations as C_matrix_ using the formula F3.

For anandamide measurements, a microdialysis catheter was placed into subcutaneous abdominal adipose tissue of two healthy, normal weight male volunteers. At a flow rate of 2 µL/min, three 240 µL microdialysis samples were collected over 6 hours for anandamide measurements. For best anandamide relative recovery, sterile solution of 10% HP-ß-CD in Ringer was chosen as perfusion fluid at a flow rate of 2 µL/min.

## Results

### In vitro Experiments

**Figure 1:**
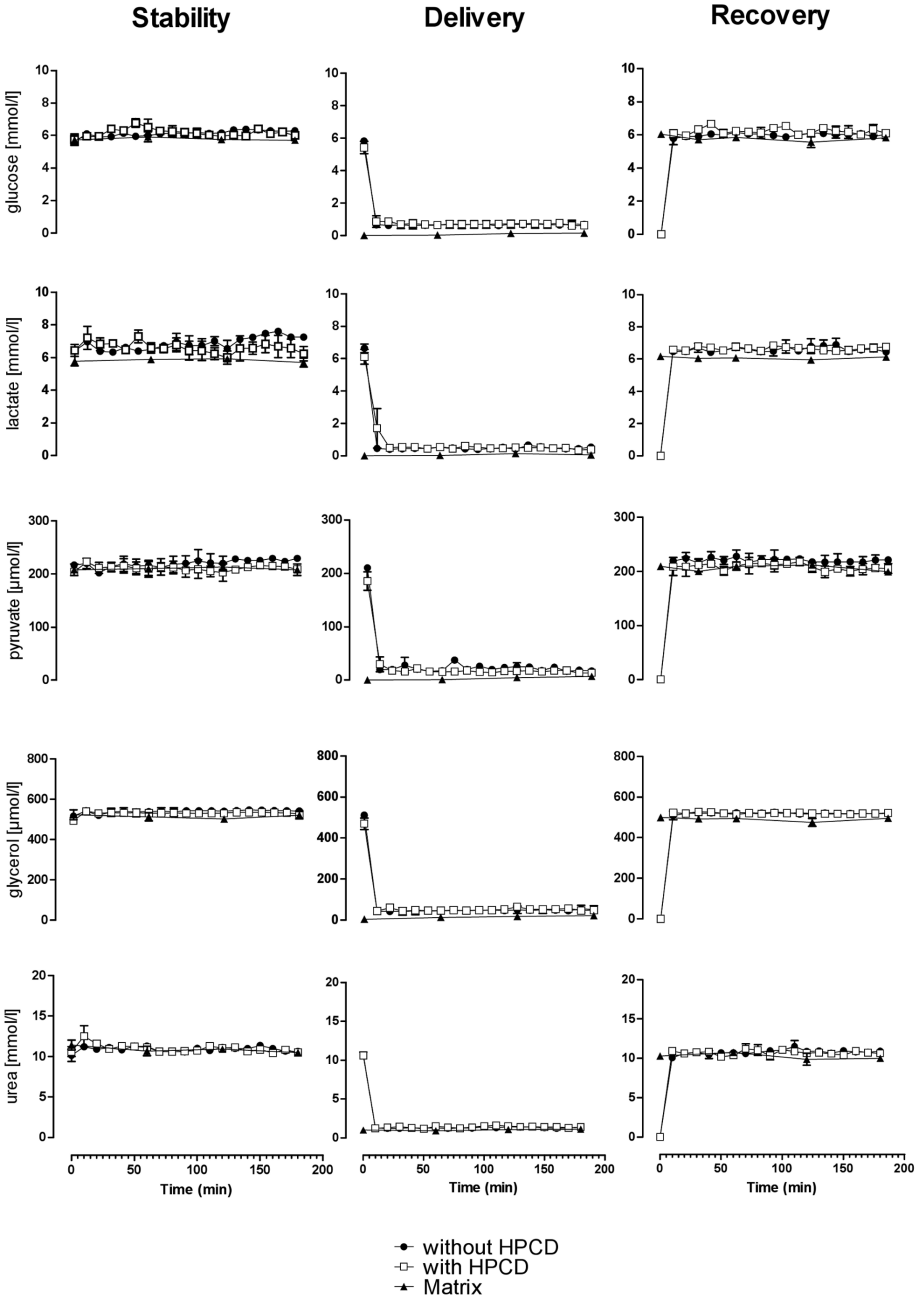
In vitro experiments assessing metabolite stability, delivery, and recovery with and without HP-ß-CD as a supplement to Ringer perfusion fluid (data: mean ± SEM).

Stability experiments showed constant metabolite concentrations in microdialysis and matrix fluid over 2 hours at 37°C independent to the use of HP-ß-CD (figure 1). Delivery experiments with a perfusion rate of 2 µL/min revealed complete equilibration for all metabolites not later than 20 min after catheter perfusion. Relative recovery in the delivery experiments ranged between 82.6±0.2% and 89.6±0.3% for perfusion fluid without HP-ß-CD and from 82.3±0.9% to 88.9±0.8% for HP-ß-CD containing perfusion fluid (table 1).

**Table 1:** In vitro metabolite delivery and recovery rates (%) at different perfusion rates (after 20 min equilibration time, mean ± SEM).

		Delivery @ 2 µL/min (n = 32)	Recovery @ 0.3 µL/min (n = 2)	Recovery @ 1 µL/min (n = 32)	Recovery @ 2 µL/min (n = 2)	Flow rate variation - goodness of fit to linear regression (r^2^)
**Glucose**	**without HP-ß-CD**	83±0.2	104±1.2	100±0.5	90±9.3	0.96
	**with HP-ß-CD**	82±0.9	113±2.3	104±0.8	98±0.4	0.95
**Lactate**	**without HP-ß-CD**	90±0.3	111±1.8	110±0.8	104±5.1	0.92
	**with HP-ß-CD**	88±1.0	117±1.8	111±0.5	105±1.8	0.99
**Pyruvate**	**without HP-ß-CD**	87±0.6	109±2.2	110±0.9	101±5.1	0.75
	**with HP-ß-CD**	89±0.8	105±2.4	104±1.0	97±3.4	0.91
**Glycerol**	**without HP-ß-CD**	85±0.3	106±0.0	104±0.2	97±2.7	0.94
	**with HP-ß-CD**	84±1.1	107±0.2	104±0.2	99±1.3	0.99
**Urea**	**without HP-ß-CD**	86±0.3	110±0.8	108±0.6	105±2.0	0.97
	**with HP-ß-CD**	84±0.8	108±2.3	107±0.8	105±0.4	0.97

Recovery experiments at a flow rate of 1 µL/min revealed stable recovery rates after 20 min equilibration time (figure 1). Relative recovery in the recovery experiments varied between 100.3±1.2% and 110.3±0.9% for perfusion fluid without HP-ß-CD and between 104.1±0.8% and 111.0±0.5 for HP-ß-CD containing perfusion fluid (table 1).

Recovery rates linearly decreased with increasing flow rates from 0.3 to 2 µL/min. Calculated coefficients of determination of the linear regressions were r^2^ = 0.75 to r^2^ = 0.99 (table 1). Maximal recovery rates at a flow rate of 0.3 µL/min were 111.4±1.8% without HP-ß-CD and 117.4±1.8% with HP-ß-CD and minimal recovery rates at a flow rate of 2 µL/min were 89.6±9.4% without and 97.0±3.4% with the use of HP-ß-CD (table 1).

### In vivo Experiments

**Figure 2:**
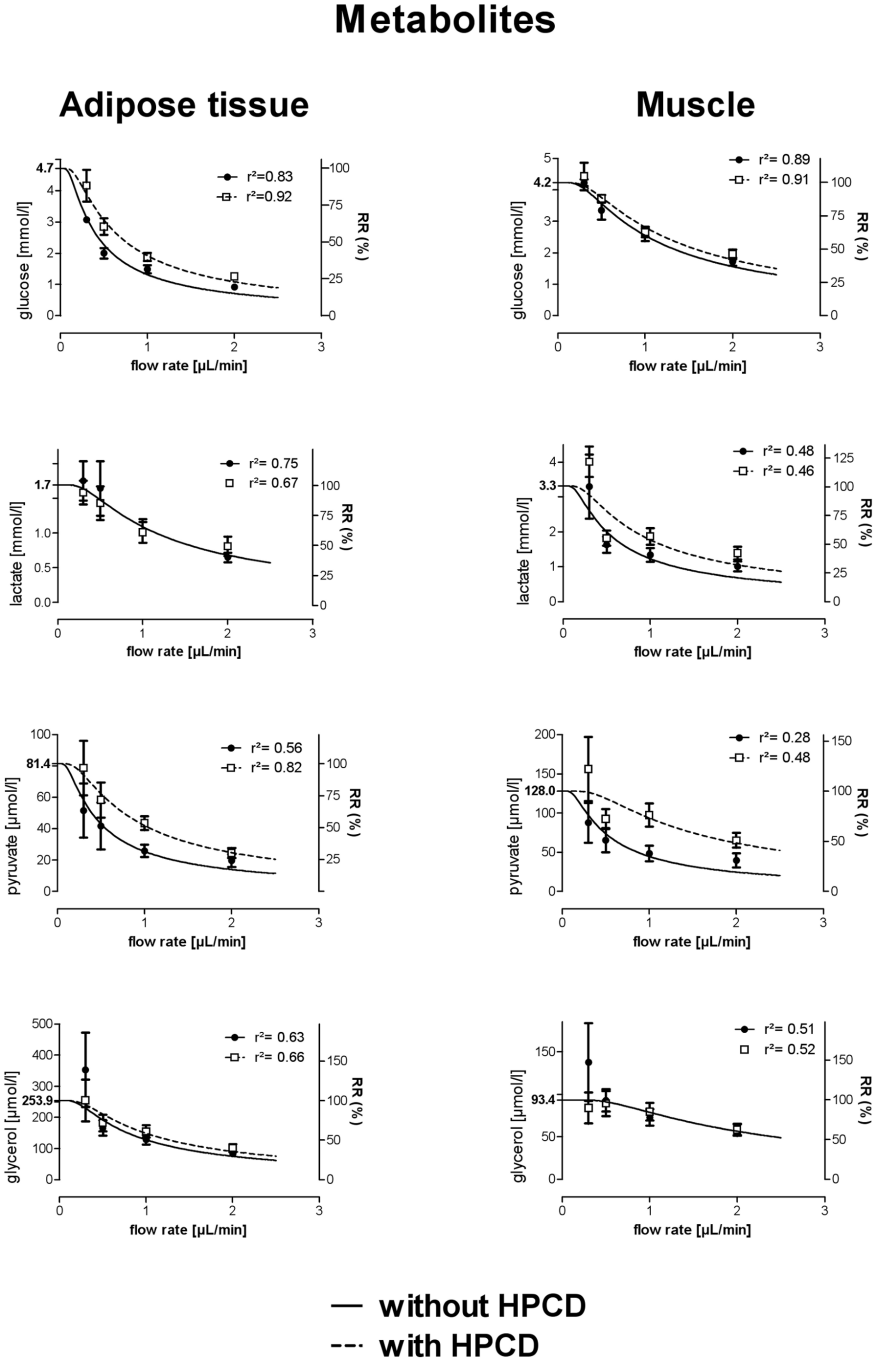
Metabolite recovery at various perfusion rates in adipose tissue and skeletal muscle of human healthy volunteers using CMA 60 catheters. Ringer perfusion fluids with and without HP-ß-CD are compared. For extrapolation of tissue concentrations, the equation C_dial_ = C_0_(1−e^−rA/F^) was used, relative recovery was calculated by RR_recovery_ = (C_microdial_/C_matrix_) x 100 (data: mean ± SEM).

**Figure 3:**
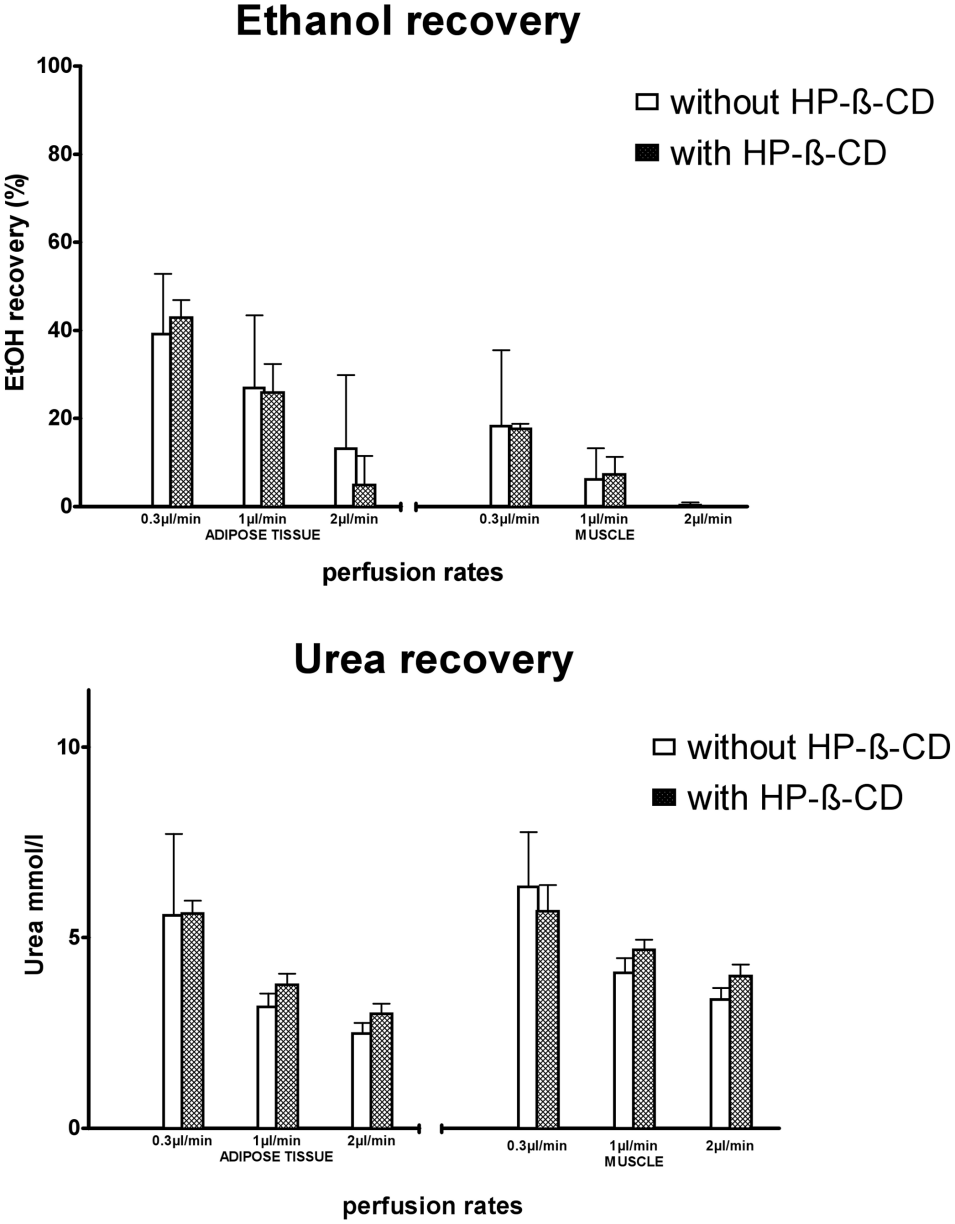
Urea and Ethanol recovery in adipose tissue and skeletal muscle Ringer perfusion fluids with and without HP-ß-CD were compared at different flow rates (data: mean ± SEM).

**Figure 4:**
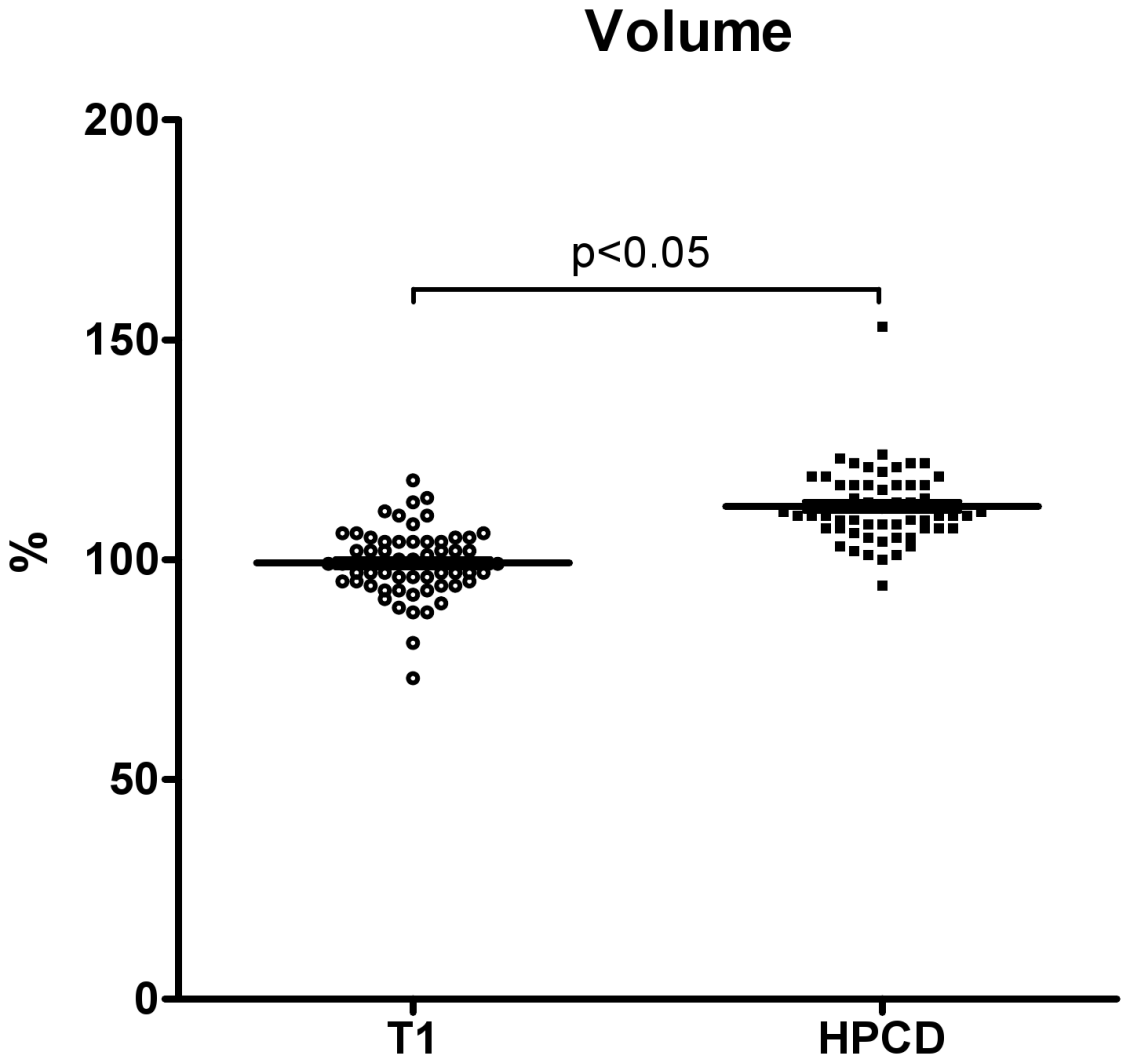
Volume escape from Ringer perfusion fluids with and without HP-ß-CD at a flow rate of 2 µL/min and a collection time of 10 minutes. 20 µl were defined as 100% (data: mean ± SEM).

Eight healthy men (28.0±2.2 years, 1.76±0.03 m, 77.5±3.6 kg) and twelve healthy women (28.0±2.1 years, 1.71±0.01 m, 73.9±2.4 kg) participated in our *in vivo* study. Glucose, lactate, pyruvate, glycerol and urea concentrations in microdialysis dialysates obtained at a flow rate of 1 µL/min were in the expected range (table 2). All metabolites were measurable in microdialysates independently of HP-ß-CD content of the perfusion fluid and without any obvious malfunction of the CMA 600 analyzer. Glucose, lactate, and pyruvate concentrations were generally higher in skeletal muscle than in adipose tissue microdialysates, whereas glycerol concentration was higher in adipose tissue dialysates (table 2). Addition of 10% HP-ß-CD to the perfusate numerically increased microdialysate metabolite concentrations (figure 2). With the exception of pyruvate, which significantly increased with HP-ß-CD, these changes were not statistically significant (table 2). For all metabolites, relative recovery was inversely related to flow rate with higher relative recovery values at lower flow rates (figure 2). Estimated interstitial values were within the expected range [17], [24], [25]. Recovery rates in proportion to the estimated interstitial values were approximately 25% at 2 µL/min and 100% at 0.3 µL/min flow rate. *In vivo* flow rate variation studies differed from *in vitro* findings. Where the latter resulted in a linear function, relative recovery rates acquired *in vivo* were not well described by a linear function. Non-linear regression of the function described by F3 lead to regression coefficients (r^2^) between 0.28 for pyruvate and 0.92 for glucose recovery. Absolute metabolite concentrations in microdialysates obtained at very low flow rates tended to be higher than estimated interstitial concentrations. The effect was most obvious for pyruvate (figure 2). Ethanol concentrations were between 15 and 25% of the originally perfusate concentration (50 nM) in skeletal muscle dialysates and between 50 and 60% in adipose tissue dialysates, independently of HP-ß-CD (figure 3). Urea microdialysate concentrations were independent of HP-ß-CD and, as for the other metabolites, relative recovery was inversely related to flow rate (figure 3). HP-ß-CD added to the perfusate increased microdialysate volume by 10% (figure 4).

**Table 2:** In vivo microdialysis concentrations at 1 µl/min flow-rate (n = 12, mean ± SEM).

	adipose tissue			skeletal muscle		
	Ringer	Ringer+ HP-ß-CD	p Value (Ringer vs. HP-ß-CD)	Ringer	Ringer+ HP-ß-CD	p Value (Ringer vs. HP-ß-CD)
**glucose (mM)**	1.4±0.1	1.8±0.2	0.10	2.6±0.2	2.7±0.2	0.73
**lactate (mM)**	1.0±0.2	1.0±0.2	0.95	1.3±0.2	1.9±0.2	0.12
**pyruvate (µM)**	27.4±4.1	43.2±4.5	0.03	48.4±9.8	89.2±14.1	0.05
**glycerol (µM)**	128.8±15.1	155.3±19.9	0.34	72.6±9.7	79.3±10.3	0.65
**urea (mM)**	3.2±0.3	3.9±0.3	0.16	4.1±0.4	4.8±0.3	0.15

**Figure 5:**
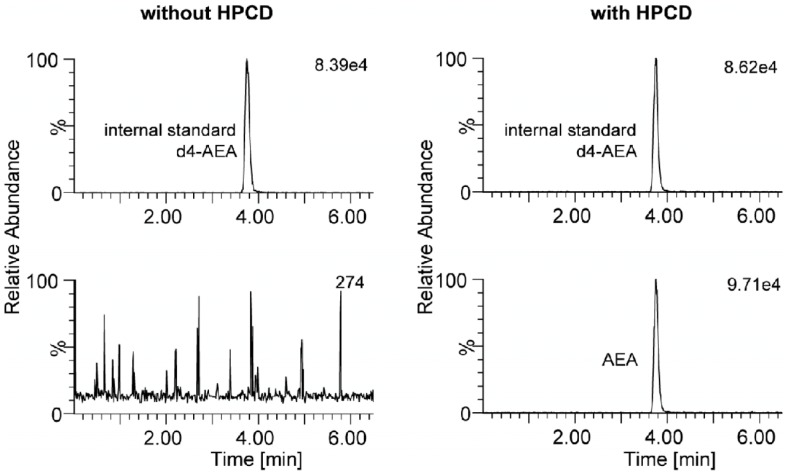
LC-MS/MS analysis of anandamide in microdialysate samples from in vivo experiments in abdominal adipose tissue. The internal standard, d4-anandamide, was spiked to the microdialysate samples. A chromatogram from dialysate obtained without HP-ß-CD is shown in the left panel. The absence of any anandamide peak (lower left panel) shows that the detected anandamide in microdialysate samples originates from endogenous sources and is not a contamination of d4-anandamide serving as the internal standard (lower right panel).

In the samples collected with CMA 60 probes in abdominal adipose tissue and HP-ß-CD supplemented perfusion fluid, anandamide was measurable (Figure 5 illustrates a representative chromatogram). Anandamide concentration was 70.2 pM ±14.7 pM (mean ± SEM) in microdialysates. Given the previously described erratic relationship between flow rate and relative recovery for anandamide [14], *in vivo* calibration using flow rate variation was impossible. Based on *in vitro* experiments, we assumed a relative recovery of 5% [14], and thus calculated *in vivo* anandamide tissue concentrations of about 3 nM.

## Discussion

The main finding of our study is that HP-ß-CD can be added to microdialysis perfusates to enhance recovery of lipophilic molecules that would otherwise not be measurable. Moreover, HP-ß-CD in a concentration of 10% (w/v) was safe and had a modest and predictable effect on glucose, lactate, pyruvate, glycerol and urea measurements. These metabolites are commonly studied metabolites in animal and human microdialysis research.

We conducted several *in vitro* experiments to evaluate possible negative influences of HP-ß-CD on analysis, stability, delivery, and recovery of glucose, lactate, pyruvate, glycerol and urea. 10% HP-ß-CD had a negligible effect on *in vitro* metabolite recovery, both, in delivery and in recovery experiments, and did not influence metabolite stability. *In vitro* recovery measurements provide insight in molecular properties of specific analytes. Yet, *in vivo* recovery can differ from from *in vitro* recovery due to mechanisms that are not fully understood. For example, analytes may bind to other molecules or surfaces that are not present *in vitro*. Whenever possible *in vivo* recovery should be determined [26]. However, these measurements can substantially prolong the experiment, which may not be tolerated by all study participants.

In our *in vivo* experiments, we assessed influences of HP-ß-CD on dialysate volume, tissue blood flow, and tissue metabolism. HP-ß-CD supplementation increased microdialysate volumes by approximately 10%. The response could be beneficial because microdialysate volumes, which are usually in the µL range, can limit analyte detection, particularly when high cut-off probes are used to detect larger molecules [27]. In fact, addition of colloids to microdialysis perfusates has previously been applied to attenuate perfusate loss into the tissue [28], [29]. Yet, metabolite concentrations may be altered in a way that is difficult to predict. Moreover, the fluid shift could directly affect tissue function or perfusion.

Tissue blood flow profoundly affects interstitial metabolite concentrations [2], [30]. We applied two complimentary methods to exclude that HP-ß-CD changes tissue perfusion in the area surrounding the microdialysis catheter, namely urea clearance and ethanol recovery [20], [21]. Since both methods rely on spectrophotometric assays, we excluded that HP-ß-CD interferes with the assays. We did not observe influences of HP-ß-CD on urea clearance or ethanol recovery suggesting that tissue blood flow was unaltered.

We assessed tissue metabolites with and without HP-ß-CD addition to the perfusate for two reasons. First, we were interested whether lipophilic molecules, requiring HP-ß-CD supplementation of the perfusate, and water soluble metabolites could be assessed using the same microdialysis catheter. Second, changes in oxidative metabolism have been recently recognized as important readout for cell toxicity [31], [32]. Lactate and pyruvate measurements in microdialysates are particularly relevant because they provide information regarding glycolysis and oxidative glucose metabolism [3], [4], [19]. Our experiments revealed tissue metabolite concentrations within the expected physiological range suggesting that HP-ß-CD is not overtly toxic. Yet, HP-ß-CD addition seems to slightly enhance metabolite recovery. Metabolite recovery depends on perfusate flow rate [23]. The relationship between recovery and flow rate was unchanged with HP-ß-CD. We observed somewhat counterintuitive recovery rates above 100% with very low perfusate flow rates. The phenomenon may be explained by evaporation of the small microdialysate sample volume (10 µL each) and occurred with and without HP-ß-CD. While the amount of HP-ß-CD delivered through the microdialysis catheter tube is minimal and even less HP-ß-CD crosses the membrane, we cannot completely rule out that HP-ß-CD causes local metabolic changes.

Our *in vitro* and *in vivo* findings suggest that HP-ß-CD could be applied as microdialysis supplement to assess interstitial concentrations of a wide range of lipophilic molecules including fatty acids, fatty acid derivatives, and phospholipids. Indeed, anandamide was only detectable in HP-ß-CD supplemented microdialysates. There is no indication that HP-ß-CD enhanced microdialysis could only be applied in specific tissues. Instead, we suggest that our approach can be used in all tissues suitable for microdialysis, both, in animal experiments and in clinical studies. Moreover, HP-ß-CD enhanced microdialysis can be applied to monitor interstitial concentrations of lipophilic drugs that may not be sufficiently recovered with standard methods. Difficulties assessing lipophilic drug concentrations in microdialysates have recently been discussed for doxorubicin [33]. Finally, the methodology may provide a new approach for comprehensive clinical investigations. Simultaneous measurements of lipophilic drugs and metabolites, such as glucose, lactate and pyruvate, provides insight in pharmacokinetics as well as pharmacodynamics or tissue toxicity [31], [32].

We conclude that addition of 10% HP-ß-CD to microdialysis perfusate solutions is a feasible and safe approach to improve recovery of lipophilic substances like endogenous fatty acid derivatives. Thus, HP-ß-CD enhanced microdialysis adds to the armamentarium required to probe the interstitial space in human subjects. Different additives have been evaluated previously to increase recovery and minimize water loss from the microdialysis probe [28], [29], [34]. HP-ß-CD is a well known molecule with good complexation properties and low toxicity used in several pharmaceutical preparations [35]. An interesting approach enabling recovery of macromolecules like bioactive peptides and proteins is using higher molecular weight cutoff microdialysis catheters. The addition of HP-ß-CD to the perfusate might be beneficial in this case [27], [36], [37]. Finally, membrane-free open-flow microperfusion is an alternative method to recover interstitial fluid [38].
